# Impact of Executive Functions and Parental Anxiety on the Development of Social Cognition in Premature Children: A Cross-Sectional Case-Control Protocol

**DOI:** 10.3389/fpsyt.2021.484571

**Published:** 2021-09-09

**Authors:** Julien Eutrope, Alexandre Novo, Coralie Barbe, Gauthier Loron, Anne-Catherine Rolland, Stéphanie Caillies

**Affiliations:** ^1^CHU Reims, Hôpital Robert Debré, Service de Psychothérapie de l'Enfant et de l'Adolescent, Reims, France; ^2^Université de Reims Champagne Ardenne, C2S EA 6291, Reims, France; ^3^Université de Reims Champagne Ardenne, Faculté de Médecine, Reims, France; ^4^Université de Paris, CRPMS ED 450, Paris, France; ^5^CHU Reims, Hôpital Maison Blanche, Unité d'Aide Méthodologique à la Recherche Clinique, Reims, France; ^6^CHU Reims, American Memorial Hospital, Service de Pédiatrie, Reims, France

**Keywords:** premature children, executive function, parental anxiety, parental depression, perinatal post-traumatic stress disorder, social cognition, theory of mind

## Abstract

**Background:** Recent research has identified neuropsychological disorders, specifically executive function disorders, in premature children. Executive functions support goal-oriented mental activity and play a role in the development of social cognition. This underlies the social and emotional behavior of individuals. Parental anxiety is also an important environmental factor that can influence the psycho-emotional development of children.

**Objectives:** The present protocol aims to compare the development of social cognition in school-age children born prematurely to that of school-age children born full-term, and to determine the impact of executive (dys)function and parental anxiety on such development.

**Methods/Design:** In this cross-sectional protocol, 28 prematurely born children aged 7-10 years (“preterm”) and 28 full-term born children aged 7-10 years (“control”) will be included. The “preterm” and “control” groups will be matched for sex and age. The neuropsychological evaluation will include that of non-verbal intellectual efficiency (Raven's colored progressive matrices), verbal level (WISC-IV subtests), and executive functions (NEPSY II subtests and the opposite worlds of TEA-CH). The evaluation of social cognition will be conducted via tests of the theory of cognitive and affective mind. Several dimensions of the level of parental anxiety will be collected through the Spielberg Trait Anxiety Inventory Form Y, Beck Depression Inventory, Social Support Questionnaire-6, Parental Stress Index and, specifically for mothers, the Modified Perinatal Post-Traumatic Stress Disorder Questionnaire.

**Discussion:** The results of this protocol will aid our understanding of the development of social cognition in premature children and to determine the factors that influence such development. This clinical research project, although following a fundamental approach, will have clinical implications because a more precise description of the development of social cognition in this school-age population will make it possible to better determine the cognitive targets of therapeutic actions and to search for predictive indices of the efficacy of practices.

**Trial Registration:**https://clinicaltrials.gov/ct2/show/NCT03007095, identifier: NCT03007095.

## Introduction

According to the World Health Organization (2013), a child born before 37 weeks of amenorrhea is considered to be premature. Prematurity is strictly defined as a birth before 37 weeks, significant prematurity before 32 weeks, and very significant prematurity before 28 weeks. In France, preterm birth affects 50,000-60,000 children every year (7.4% of live births in 2010 (1). In almost all countries with reliable data, premature birth rates have been increasing for several years. This finding necessitates the study of the development and future of premature children. The literature indicates that such children are more likely to have cognitive and motor disorders, behavioral disorders, and academic problems than are full-term (2) children, with the severity of the disorders largely dependent on gestational age and/or birth weight (3).

In recent years, research has identified neuropsychological disorders, specifically executive function disorders, in premature children, even in so-called “late premature children.” Executive functions are mental processes that are goal-oriented and allow for an adaptive response to the environment. Three sets of executive functions are generally considered (4): inhibition, working memory, and cognitive flexibility. Inhibition refers to the ability to resist and not act impulsively, thereby integrating attentional control. Working memory refers to the ability to mentally maintain and process information. Flexibility is the ability to alter one's perspective in response to changes in the environment and, in a complementary manner, to maintain a mental perspective when the environment is irrelevant. Some authors describe these executive functions (4, 5) as basic elements necessary for the implementation of other so-called superior executive functions: reasoning, problem solving and planning (4–6). A growing body of research indicates the difficulties experienced by premature children in tasks that require such executive functions (7). It should be noted, however, that this research is not consensual as to which executive functions are most affected by the degree of prematurity. Indeed, while some authors observed a lack of cognitive flexibility, working memory, and inhibition (8) in very significantly premature children aged 4–12 years compared to that in children born full-term, other authors found differences only in cognitive flexibility between very significantly premature children aged 2 years old (9) or 8–12 years old (10) and children born full-term, without any impact on working memory and inhibition.

The impact of such executive dysfunction on the development of premature children is poorly understood. Few studies have related this dysfunction to academic performance (11–15), and no research has linked such executive dysfunction to social cognition. Social cognition is the study of the cognitive processes that underlie the social and emotional behavior of individuals (16). Social cognition thus integrates different skills, including the ability to understand the emotional and cognitive mental states of others, which are known as the affective and cognitive theories of mind. The literature on the development of full-term children highlights the role of executive functions in the development of these theories of mind, both cognitive (17) and affective (18). In large syntheses such as those by Schneider et al. (19) or Nader-Grosbois et al. (20) hypothesize links between the development of executive functions such as auditory attention, inhibition or shifting and the construction of theories of mind (notably affective with false beliefs). It is therefore prudent to query whether premature children, because of their executive dysfunction, atypically develop the theories of the mind, which is a key concept in social cognition. There is little literature on this subject, despite children born very significantly prematurely often being described as having difficulties in social relations (21). Only the study by Jones et al. (22) attempted to answer this question, but did not yield any convincing results with regard to the cognitive theory of mind.

Finally, the anxiety level of the mother and, more broadly, of the parents is known to impact the development of the premature child (23). According to Guralnick (24), the premature child would be exposed to the “double-stress” of parental care and anxiety and would therefore be twice as vulnerable. The child's developmental trajectory would then endure the direct effects of neurodevelopmental immaturity and the indirect effects linked to the tryptic stress/anxiety/depression mediated by daily interactions with parents. Singer (25) and Pierrehumbert (26) confirmed this hypothesis and highlighted a correlation between maternal psychopathologies (anxiety, depression) and the child's state of health. It therefore seems essential to consider the effect of this contextual aspect on the development of the child's social cognition.

## Objectives

### Main Objective

The main objective of this protocol is to compare the development of the theories of mind (affective and cognitive) in school-age children born prematurely to those of school-age children born full-term.

### Secondary Objectives

The secondary objectives will be (1) to study the association between the executive functioning and theories of mind of school-age children and (2) to study the association between parental anxiety and theories of mind of school-age children.

## Hypotheses

A developmental delay or deficit in the theory of mind in school-age children born prematurely compared to that in school-age children born full-term is expected. This delay or deficit is thought to be related to executive dysfunction and/or the level of parental anxiety.

## Methods

### Ethical Approval

This protocol was approved by the Ethics Committee of Nancy Est III (12/06/2016) and by the French Agency for Medicines and Health Products (08/18/2016). The protocol will be conducted according to the principles of the Declaration of Helsinki and in line with the Guidelines for Good Clinical Practice.

### Protocol Design

This is a cross-sectional protocol of the preterm/control type.

### Participants and Procedure for Recruitment

The subjects will be children born at the maternity ward of the CHU de Reims, France (Reims University Hospital). Information will be given by telephone to caretakers by a clinical research assistant. Once the parent or legal guardian of the children consent to participate in the study, written informed consent will be obtained from the parent or legal guardian of the children. Both the child and the parents or legal guardian must provide their consent for the child's participation in the study.

A total of 28 children born prematurely at the Reims University Hospital, aged 7-10 years (“preterm”), and 28 children born full-term at the Reims University Hospital, aged 7-10 years (“control”), will be included. The “preterm” and “control” groups will be matched for sex and age (± 3 months).

The inclusion criteria are as follows:

“Preterm” children: (1) Children born prematurely (before 37 weeks), making a clear distinction between high and medium preterm infants, (2) aged 7–10 years at the time of inclusion, and (3) enrolled in a non-specialized primary school;

“Control” (full-term) children: (1) Children born at ≥37 weeks, (2) aged 7–10 years, and (3) enrolled in primary school.

“Preterm” and “Control” children: (1) Children and parents affiliated with a social security system, and (2) holders of parental authority will have signed an informed consent form.

The exclusion criteria for the “preterm” and “control” children are as follows:

*Related to the child:* (1) Children with intrauterine growth restriction, (2) multi-pregnancy children, (3) children with organic malformation, (4) children with genetic abnormalities, (5) children with neuro-motor pathology, (6) children with overall developmental delay (IQ of <80), and (7) children refusing to participate in the study.

*Related to the parents:* (1) Minor parents, (2) parents with a proven intellectual deficit, (3) parents with acute or chronic psychotic disorders, (4) parents with difficulties in understanding and speaking the French language, (5) parents under a protective regime (guardianship or curatorship), and (6) parents refusing to participate in the study.

### Study Interventions

As shown in Table 1, participation in the study will consist of two visits by psychologists or psychiatrists trained at various assessment scales. Data collection for parents will be carried out during the first visit. Data collection for children will be carried out over the two visits. The adoption of two visits for the children will allow for better conditions for the passing of the different evaluation scales and will also improve the children's comfort.

**Table 1:** Protocol design.

	**Visit 1**	**Visit 2**
**Enrollment**		
Eligibility screen	X	
Informed consent	X	
Clinical data record	X	
**Caretakers evaluation**		
STAI: state-trait anxiety inventory	X	
BDI: beck depression inventory	X	
PSSQ: perceived social support questionnaire	X	
mPPQ: modified perinatal post-traumatic stress disorder questionnaire	X	
PSI: parental stress index	X	
**Child evaluation (“premature” and “control”)**		
Vocabulary (WISC-IV)	X	
Similarities (WISC-IV)	X	
Same and opposite worlds (TEA-CH)	X	
Auditory attention (NEPSY-II)	X	
Cognitive theory of mind	X	
Raven's colored progressive matrices		X
Repetition of sentences (NEPSY-II)		X
Affective theory of mind		X

### Outcome Measurements

#### Neuropsychological Evaluation of Exposed and Unexposed Children

The neuropsychological evaluation will analyze the children's non-verbal intellectual efficiency, their verbal level, and their executive functions (attention, inhibition, flexibility, and working memory).

##### Raven's Colored Progressive Matrices

This test evaluates non-verbal intellectual efficiency by measuring reasoning capacity by analogy. It has the advantage of not being sensitive to socio-cultural elements within the same ethnic group. The color version for children (27) has 36 questions grouped into 3 sets of 12 questions each, of increasing complexity.

##### Verbal Subtests of the WISC-IV

The “similarities” and “vocabulary” subtests of the WISC-IV (28) allow for an extrapolated verbal comprehension index (eVCI) to be calculated. This choice entails to compute a prorata because we only want to assess semantic competences, and not social cognition (or more pragmatic) competences as required by Comprehension subtest. We propose this method in order to focus only on semantic skills competences and to avoid a possible bias in the evaluation of the real skills of the two populations studied in theory of mind assessed through these semantic comptences. The “similarities” subtest includes 23 pairs of words of increasing difficulty and specifically evaluates verbal abstraction. The “vocabulary” subtest contains 24 questions of increasing difficulty for which children must provide a definition.

##### Executive Functions

Auditory attention and repetition of phrases. Using two 3 min word listening tasks, the “Auditory Attention and Associated Responses” subtest of the NEuroPSYchological Assessment 2nd Edition (NEPSY-II) (29) evaluates selective auditory attention and the ability to maintain it, and the ability to modify and maintain a new rule.

The “sentence repetition” subtest evaluates the child's ability to repeat sentences of increasing complexity and length (17 in total). It allows to assess the verbal working memory capacities.

We preferred the NEPSY auditory attention test to the TEA-CH (Elevator counting) because the NEPSY assesses selective auditory attention as well as higher-level executive components that the TEA-CH does not offer.

Inhibition/flexibility. This task from the Test of Everyday Attention for Children (TEA-CH) consists of two tests and evaluates the inhibition and/or cognitive flexibility skills of a child's verbal response (30). In the first test, the “Same World,“ the child must state as quickly as possible the numbers (1 or 2) as they succeed each other in each box of a course provided on a sheet of paper. In the second test, the “Opposite World,” the child must state ”1“ when he sees a ”2“ and state ”2“ when he sees a ”1“ as quickly as possible throughout the course. Four worlds will follow in succession; one same world, two opposite worlds, and finally one same world.

#### Experimental Evaluation of Social Cognition in Exposed and Unexposed Children

Theory of Mind tests are not standardized for the moment, neither in English or French, except for the very simple theory of mind item of the NEPSY-2. Literature on theory of mind is currently based on an experimental approach rather than on a standardized test approach. Our theory of mind assessment is adapted from previous studies. Because of the multiplicity of terms in the field of social cognition – theory of mind implicit, explicit, cognitive, affective- and knowing that it does not cover exactly the same functions, we are interested in what happens between 7 and 10 years old when it comes to articulating the mental states of belief (false belief) or non-intention (false step) to emotions.

##### Cognitive Theory of Mind Tasks

The cognitive theory of mind is evaluated using the concept of false belief, which is accepted as a reliable indicator. Four stories of false beliefs, based on published tasks (31–34), are told to children using illustrations. Each aims to evaluate, using questions regarding the story, the children's ability to understand that a character may misconceive reality or understand what another character believes.

##### Affective Theory of Mind Tasks

Two tasks evaluating the affective theory of mind have been proposed in the form of stories told to children. The first, called an emotional perspective task (35) (based on the work of Harwood and Farrar, 2006), evaluates children's ability to understand the emotional state (joy, anger, fear, or sadness) of another even when the other person experiences an emotion that is different from their own. The second, called the emotion inference task (36), measures children's ability to deduce (1) the emotion experienced by a character (joy, anger, fear, or sadness) from his false belief (he is sad because his belief is false) but also (2) the emotion experienced by a character who is a victim of a faux pas (37).

#### Clinical Data Collected From Parents

##### The State-Trait Anxiety Inventory Scale Version Y

The State-Trait Anxiety Inventory (STAI) (38) is designed to evaluate anxiety-trait and anxiety-state using 20 questions that only concern the psychological and non-somatic aspects of anxiety. Version Y was developed to eliminate questions that are more related to depression. The STAI is used both in practice and in clinical research. It includes separate scales for evaluating the state (STAI Form Y-A) and the trait (STAI Form Y-B). Each of the scales includes 20 propositions; the S scale to evaluate what subjects are feeling at the time, and the T scale to capture what subjects are generally feeling. The STAI is intended for self-administration, and can be completed individually or in groups. The handover time is approximately 10 min to complete both scales. Each response to a proposition in the questionnaire corresponds to a score of 1 to 4 (1 indicating the lowest level of anxiety and 4 indicating the highest level).

##### The Beck Depression Inventory Scale, 2nd Version

The Beck Depression Inventory 2nd version (BDI-II) scale (39) has three objectives; to screen for depression, to evaluate the severity of previously diagnosed depression, and to aid monitoring of the efficacy of therapeutic interventions. The tool provides a quantitative estimate of the intensity of depressive feelings. It includes 21 questions of symptoms and attitudes that describe a specific behavioral manifestation of depression, graduating from 0 to 3 using a series of for statements reflecting the severity of the symptoms. This scale was translated and validated to French by Bourque and Beaudette in 1982 (40).

##### Perceived Social Support Questionnaire-6

The Perceived Social Support Questionnaire (PSSQ) (41) is a self-evaluation scale that evaluates the availability of and satisfaction with perceived social support. The authors were inspired by Bowlby's attachment theory, associating the notion of social support with basic needs; the need for closeness to the mother, and the need for a meaningful privileged relationship with others. This scale therefore makes it possible to evaluate the type of support received, the sources of this support, the number of people who provide it (or availability), and the perceived quality (or satisfaction). The four main forms of social support are represented by the four questions of the scale; esteem support (comfort, listening in difficult times), material or financial support (direct assistance when necessary), informational support (advice or suggestions from others), and emotional support (reassurance, confidence building). For each type of support, the questionnaire makes it possible to know how many people provide it, who these people are (family, friends, colleagues, specialists, etc.), and whether the subject is satisfied with this support. Two scores are obtained for each subject; availability (number of people who participated in the support) and perceived satisfaction (”quality“) of the support. This tool also provides information regarding the nature of the person's perceived social support. This is a self-administered questionnaire completed by the subject (requiring 4–6 min to complete). Regarding the four questions related to the availability of social support, the subject must indicate the number of people they can rely on in these four categories; family, friends/peers, colleagues, and health professionals. For the satisfaction questions, the subjects are required to rate their level of satisfaction on a five-point Likert scale. The total availability score is obtained by summing the responses indicated by the subject on the ”how many“ questions. To calculate the total satisfaction score for support, the responses (from 1 to 5) indicated by the subject on the satisfaction questions are summed. The questionnaire was translated to French by Bruchon-Schweitzer and Quintard (42).

##### Modified Perinatal Post-traumatic Stress Disorder Questionnaire

This is a 14-question self-administered questionnaire specially adapted for the parents of children at high perinatal risk and used to evaluate the presence of traumatic elements related to childbirth (43). Its construction refers to the DSM-IV criteria for post-traumatic stress disorder (intrusion, avoidance, and hypervigilance). The questions are retrospective, and the mother must answer questions regarding symptoms that appeared at birth and persisted for >1 month. We will use the version modified in 2006, with the same questions as in the original version (44), but with the possibility of answering on a four-point Likert scale, from 0 (not at all) to 4 (often for >1 month). The Perinatal Post-Traumatic Stress Disorder Questionnaire is specific to the birth of a baby at risk, and can be expected to be more appropriate than other scales for this particular event. This tool has been translated into French and validated by Pierrehumbert's team (45).

##### Brief Form of the Parental Stress Index

The Parental Stress Index (46) is a measurement tool used to detect difficulties in parent-child dyad interactions. Parental stress is defined as a state of psychological discomfort related to the specific area of the child's upbringing, i.e.,the stress of the parent at the time of raising the child. The questionnaire used to measure this stress consists of 101 questions divided into two categories of stressors; those associated with the child's domain and those associated with the parent's domain. The parent's responses are provided on a five-point Likert scale that represents the respondent's level of agreement or disagreement with each of the stated propositions.

The short form is comprised of 36 questions derived from the long form (101 questions). For the short form, the results are organized into three sub-scales (parental distress, dysfunction in parent-child interaction, and child difficulties) in addition to providing a total score for the stress generated by the parent-child relationship. The higher the value identified, the higher the level of stress.

#### Socio-Demographic Data

##### Clinical Data of Pregnancy

Medical assistance for procreation, risk of preterm delivery, hospitalization, pregnancy-related pathologies, intercurrent events during pregnancy, and type of delivery (normal, or scheduled or emergency cesarean section).

##### For the Child

Age, sex, school level, birth in weeks of amenorrhea, gestational weight, rank in siblings, childcare, breastfeeding, medical/surgical history, APGAR and Perinatal Risk Inventory (PRI) score, specific follow-up (speech therapy, psychomotricity, orthoptics, psychological follow-up), and child psychiatry or neuropediatric monitoring.

##### For the Parents

Age, marital status, level of education, occupation, number of children, use of psychoactive substances (alcohol, tobacco, cannabis, etc.), personal and family psychiatric history, and psychiatric/psychological follow-up (psychiatric hospitalization, psychotropic treatments), neurological history (follow-up, treatment).

##### For the Mother

Pregnancy, parity, and miscarriages (number, trimesters, etc.).

#### Sample Size

Because of lack of literature, sample size calculation could not be performed on association between theories of mind and prematurity. Because of link between verbal skills and TOM has been shown in several studies that highlighted the impact of verbal skills on TOM task performance (47, 48), the calculation of the number of subjects required was performed for association between prematurity and WISC VCI with the following hypotheses: an average VCI score for “control” patients of 103.2 ± 12.6 (49) and an average VCI score for “preterm” patients of 92. With an alpha risk of 5%, a power of 90%, and a bilateral test, the number of patients required was 28 per group (NQuery software version 4.0).

#### Data Management

The study will make use of two separated databases for storing research data and contact information. The contact information of patients, such as the participant's name, phone number, and address, will be stored in a separate database unrelated to the research database. In the research database, all participants will receive an ID number. Electronic data will be saved on a secure server with restricted access. In accordance with the national regulations in France, the data will be stored for 15 years after termination of the trial.

#### Statistical Analysis

Descriptive analysis will be performed. Quantitative variables will be expressed as the mean± standard deviation or median (range) and qualitative variables as number (percentage).

Factors associated with development of the affective and cognitive theories of mind separately, notably groups (“preterm”/“full-term”) and parental anxiety, will be studied using univariate analysis (Wilcoxon tests for qualitative variables and Spearman's correlation tests for quantitative variables) and multivariate analysis (linear regression with variables with a *p* < 0.10 by univariate analysis included).

The association between executive functions, evaluated using NEPSY-II, and the development of theories of mind will be studied using univariate analysis (Wilcoxon tests for qualitative variables and Spearman's correlation tests for quantitative variables) and multivariate analysis (linear regression with variables with a *p* < 0.10 by univariate analysis included).

The association between the executive functions, evaluated using the TEA-CH, and the development of the theories of mind will be studied using univariate analysis (Wilcoxon tests for qualitative variables and Spearman's correlation tests for quantitative variables) and multivariate analysis (linear regression with variables with a *p* < 0.10 by univariate analysis included).

A *p* < 0.05 will be considered significant. Statistical analysis will be performed using SAS (Version 9.4, SAS Institute, Cary, NC, USA).

## Discussion

This research will aid the identification of the development of social cognition in premature children and potentially determine the factors that influence such development. It will therefore allow for a better understanding of the possible developmental consequences of prematurity. This clinical research project, while following a fundamental approach, will have applied implications. While many studies have documented the relationship between executive functions and the cognitive theory of mind, very few have understood the relationship between executive functions and the affective theory of mind, let alone in children aged 7-10 years. A longitudinal study by Witt et al. (50) investigates delayed theory of mind development in prematurely born children. As demonstrated by the authors, children born prematurely have overcome their difficulties in ToM around five years old. But compared to this one, our main task specifically measures emotional inference, based on a false belief or a false step. Our goal is to understand the possible difficulties of preterm infants in inferring emotions from the “cognitive” mental states of the characters. In our study, we are interested in what happens later (between seven and ten years old when it comes to articulating the mental states of belief (false belief) or non-intention (false step) to emotions, which to our knowledge has not yet been explored in this population. If there is no direct benefit for the protocol participants, we will nevertheless be able to provide a more accurate description of the development of social cognition in this school-age population. We will attempt to identify environmental and parental factors that may influence the development of social cognition and executive functions in premature children. The knowledge acquired will help to better target possible therapeutic actions in the follow-up of premature children, and better accompany and support parents affected by their child's premature birth. Subsequently, the first avenues of care that may be opened up and the further studies carried out will make it possible to evaluate their relevance and efficiency.

## Ethics Statement

This protocol was approved by the Ethics Committee of Nancy Est III (12/06/2016) and by the French Agency for Medicines and Health Products (08/18/2016). The protocol will be conducted according to the principles of the Declaration of Helsinki and in line with the Guidelines for Good Clinical Practice. Written informed consent to participate in this study was provided by the participants' legal guardian/next of kin.

## Author Contributions

JE, SC, and A-CR conceived of the protocol, performed the literature search, and conceived of the initial protocol, with the contributions of CB (reagents/materials/analysis tools). AN contributed to the design of the final protocol. JE, SC, CB, AN, and GL wrote the paper. All authors contributed to the article and approved the submitted version.

## Funding

This protocol was funded by the grant Projet Hospitalo-Universitaire Cognition Sociale et Maladies NeuroDéveloppementales from the Center Hospitalier Universitaire de Reims and Université Reims Champagne-Ardenne. The funding source had no role in the design of this protocol and will not have any role during its execution, analysis, interpretation of the data, or decision to submit results.

## Conflict of Interest

The authors declare that the research was conducted in the absence of any commercial or financial relationships that could be construed as a potential conflict of interest.

## Publisher's Note

All claims expressed in this article are solely those of the authors and do not necessarily represent those of their affiliated organizations, or those of the publisher, the editors and the reviewers. Any product that may be evaluated in this article, or claim that may be made by its manufacturer, is not guaranteed or endorsed by the publisher.

## Abbreviations

WISC IV: Wechsler Intelligence Scale for Children IVth edition
4: Verbal Comprehension Index
NEPSY-II: NEuroPSYchological Assessment 2nd Edition
TEA-CH: Test of Everyday Attention for Children
STAI: State-Trait Anxiety Inventory
BDI-II: Beck Depression Inventory 2nd version
PSSQ: Perceived Social Support Questionnaire
mPPQ: Modified Perinatal Post-Traumatic Stress Disorder Questionnaire
PSI: Parental Stress Index.
